# Supplementary material for: The causal relationship between the human gut microbiota and pyogenic arthritis: a Mendelian randomization study
Source: Front Cell Infect Microbiol. 2024 Nov 26;14:1452480. doi: 10.3389/fcimb.2024.1452480 (PMC11629706; doi:10.3389/fcimb.2024.1452480)
Supplement: Supplementary file 1 [file DataSheet1.zip › mendelian test/ebi-a-GCST90027735.csv_leave_one_out.pdf]

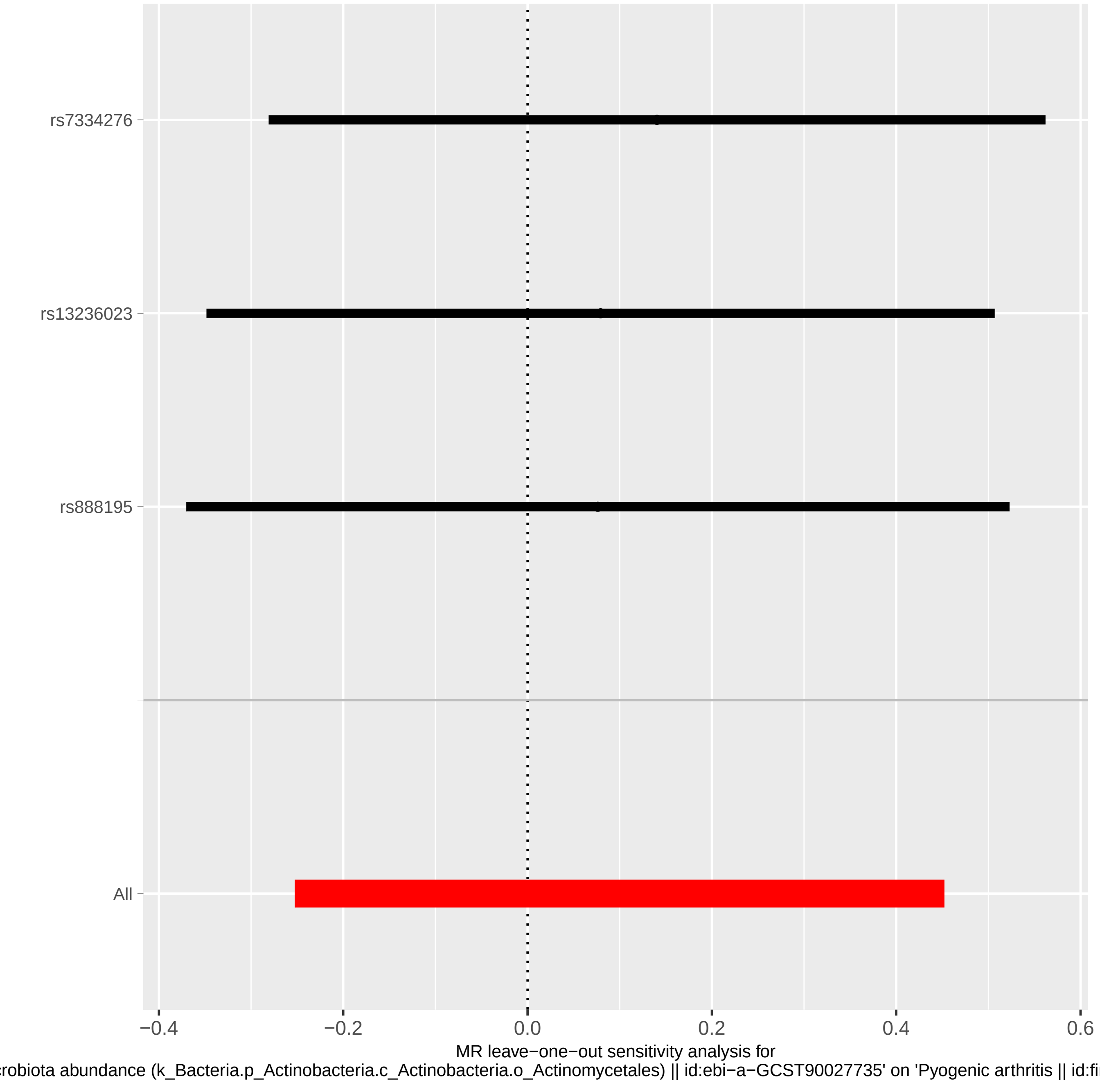
rs7334276
rs13236023
rs888195
All
−0.4
−0.2
0.0
0.2
0.4
0.6
MR leave−one−out sensitivity analysis for
crobiota abundance (k_Bacteria.p_Actinobacteria.c_Actinobacteria.o_Actinomycetales) || id:ebi−a−GCST90027735' on 'Pyogenic arthritis || id:fi
